# Edaravone suppresses retinal ganglion cell death in a mouse model of normal tension glaucoma

**DOI:** 10.1038/cddis.2017.341

**Published:** 2017-07-13

**Authors:** Kei Akaiwa, Kazuhiko Namekata, Yuriko Azuchi, Xiaoli Guo, Atsuko Kimura, Chikako Harada, Yoshinori Mitamura, Takayuki Harada

**Affiliations:** 1 Visual Research Project, Tokyo Metropolitan Institute of Medical Science, Tokyo, Japan; 2 Department of Ophthalmology, Institute of Health Biosciences, The University of Tokushima Graduate School, Tokushima, Japan

## Abstract

Glaucoma, one of the leading causes of irreversible blindness, is characterized by progressive degeneration of optic nerves and retinal ganglion cells (RGCs). In the mammalian retina, excitatory amino-acid carrier 1 (EAAC1) is expressed in neural cells, including RGCs. Loss of EAAC1 leads to RGC degeneration without elevated intraocular pressure (IOP) and exhibits glaucomatous pathology including glutamate neurotoxicity and oxidative stress. In the present study, we found that edaravone, a free radical scavenger that is used for treatment of acute brain infarction and amyotrophic lateral sclerosis (ALS), reduces oxidative stress and prevents RGC death and thinning of the inner retinal layer in EAAC1-deficient (KO) mice. In addition, *in vivo* electrophysiological analyses demonstrated that visual impairment in EAAC1 KO mice was ameliorated with edaravone treatment, clearly establishing that edaravone beneficially affects both histological and functional aspects of the glaucomatous retina. Our findings raise intriguing possibilities for the management of glaucoma by utilizing a widely prescribed drug for the treatment of acute brain infarction and ALS, edaravone, in combination with conventional treatments to lower IOP.

Glaucoma is a progressive neurodegenerative disease of the eye and the second leading cause of world blindness after cataracts.^1^ It is estimated that glaucoma will affect ~80 million individuals worldwide by 2020, with 11.1 million individuals becoming bilaterally blind.^2^ Glaucoma is caused by multiple factors and is characterized by progressive degeneration of retinal ganglion cells (RGCs) and their axons, together with visual field defects which are usually associated with elevated intraocular pressure (IOP).^3^ Normal tension glaucoma (NTG) is a subtype of glaucoma that presents with statistically normal IOP. The prevalence of NTG is reported to be higher among the Japanese than Caucasians.^4^ However, even when lowering elevated IOP, the major risk factor for the progression of glaucoma, disease can be progressed.^5^ These findings suggest a possibility that non-IOP-dependent factors may contribute to disease progression of glaucoma, especially in the context of NTG.^4, 6^

We previously reported that loss of glutamate transporters (excitatory amino-acid carrier 1 (EAAC1) or glutamate/aspartate transporter (GLAST)) in mice leads to progressive RGC loss and optic nerve degeneration without high IOP, which is similar to NTG.^7^ EAAC1 is expressed on ganglion cells and transport cysteine, as well as glutamate, into RGCs as a precursor for neuronal glutathione synthesis.^8^ Glutathione, an antioxidant, protects cells from reactive oxygen species (ROS) such as free radicals and peroxides.^9^ Thus, RGC loss in EAAC1 knockout (KO) mice is partly due to decreased glutathione synthesis leading to increased oxidative stress through the formation of ROS.^7, 10^

Edaravone is a free radical scavenger that involves electron donation to free radicals.^11^ Donation of an electron to a lipid peroxyl radical converts edaravone anion to the edaravone radical, which is oxidized to produce 3-methyl-1-phenyl-2-pyrazolin-4,5-dione and its hydrolysate, 2-oxo-3-(phenylhydrazono) butanoic acid.^12^ Edaravone quenches hydroxyl radicals (˙OH) and inhibits lipid peroxidation dependent and independent of ˙OH.^12, 13^ In an *in vitro* study using RGC-5, a cell culture model to study the neurobiology of RGCs, edaravone scavenged the intracellular ˙OH, superoxide anion (O_2_^−^˙) and hydrogen peroxide (H_2_O_2_), and it showed stronger scavenging activity against ˙OH than others.^14^ An ophthalmic study showed that edaravone attenuates retinal ischemia/reperfusion injury in rats^15^ and retinal damage in experimental high IOP glaucoma mice.^16^ These findings suggest a possibility that edaravone protects RGCs through ROS scavenging effects.

In Japan, edaravone was first approved for the treatment of acute brain infarction from 2001. A multicenter, randomized, placebo-controlled, double-blind study on acute ischemic stroke patients showed a significant improvement in functional outcome in the edaravone group as compared with the placebo group.^17^ Next, clinical trials were conducted for its use in amyotrophic lateral sclerosis (ALS) and showed that edaravone suppressed the progression of motor dysfunction without clinically significant adverse drug reactions. The level of 3-nitrotyrosine, a marker for oxidative stress, in cerebrospinal fluid was lower after edaravone treatment in almost all the patients, suggesting that edaravone could protect neuronal cells from oxidative stress.^18^ In response to these results, edaravone has been used clinically to treat ALS since 2015.

In the present study, we examined the effects of daily edaravone administration on NTG-like retinal degeneration in EAAC1 KO mice, in order to determine if edaravone is effective for treatment of glaucoma.

## Results

### Edaravone protects RGCs in EAAC1 KO mice

**Figure 1:**
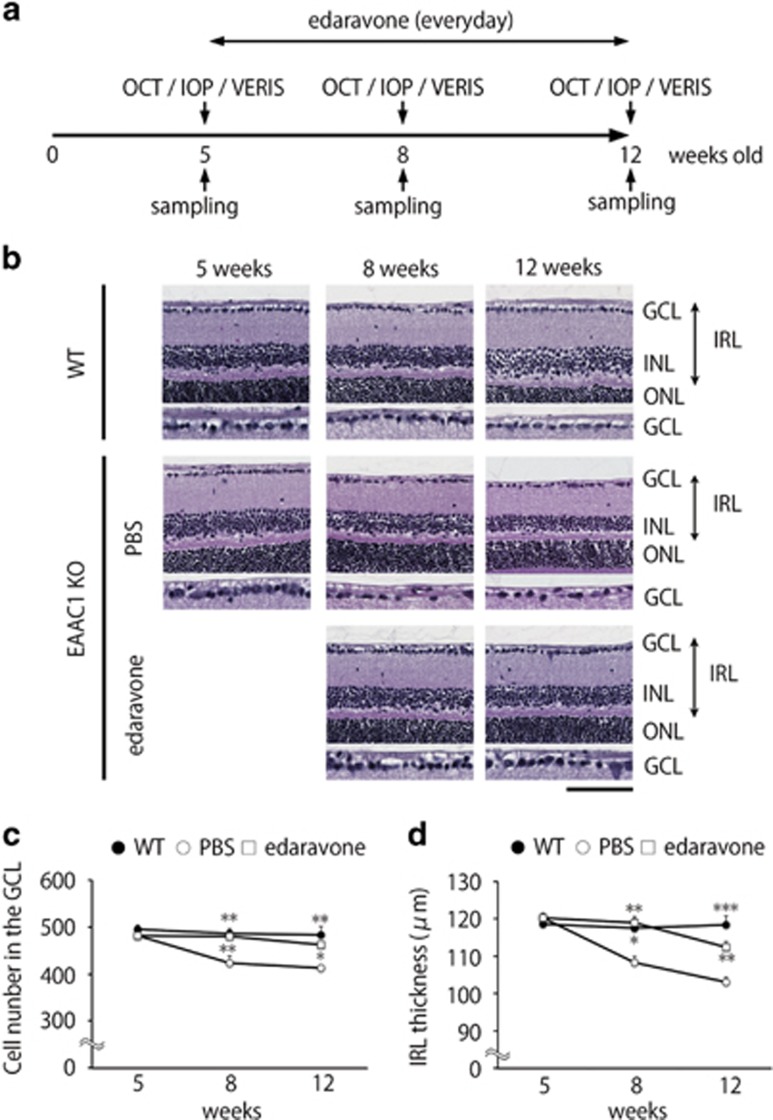
Effects of edaravone on retinal degeneration in EAAC1 KO mice. (**a**) Experimental protocols. Edaravone (3 mg/kg) or PBS was injected intraperitoneally everyday from 5 W. The mice were killed at 5, 8 and 12 W. (**b**) H&E staining of retinal sections. Scale bar, 100 and 50 *μ*m in the upper and immediately lower panels, respectively. GCL, ganglion cell layer; INL, inner nuclear layer; ONL, outer nuclear layer; IRL, inner retinal layer. (**c**, **d**) Quantification of the cell number in the GCL (**c**) and IRL thickness (**d**). The data are presented as means±S.E.M. of six samples for each experiment. *P<0.05, **P<0.01, ***P<0.001

**Figure 2:**
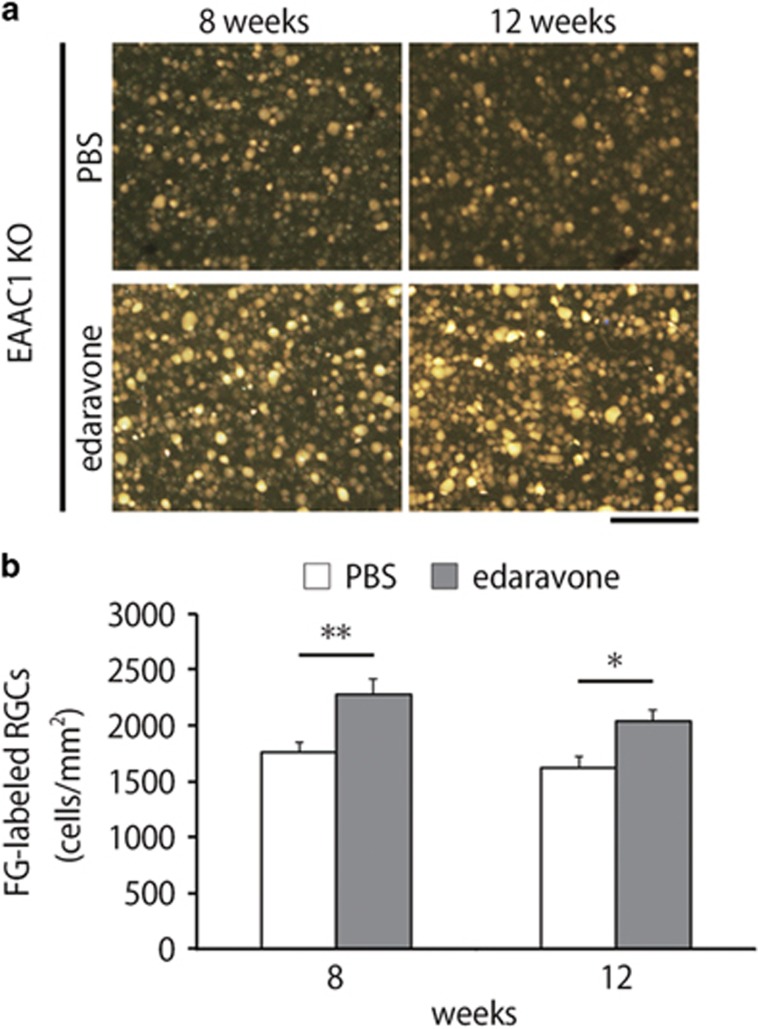
Effects of edaravone on RGC degeneration in EAAC1 KO mice. (**a**) Representative images of retrograde-labeled RGCs at 8 and 12 W. Scale bar: 100 *μ*m. (**b**) Quantitative analyses of (**a**). The data are presented as means±S.E.M. of six samples for each experiment. *P<0.05, **P<0.01

To investigate whether edaravone is capable of preventing the NTG-like phenotypes in EAAC1 KO mice, we administered edaravone or PBS (control) intraperitoneally everyday to EAAC1 KO mice from 5 weeks of age (5 W) to 8 or 12 W (Figure 1a). The retinas of EAAC1 KO mice show normal organization at 5 W, but RGC loss and the thinning in the inner retina was clear at 8 and 12 W (Figure 1b).^7, 19, 20, 21^ The cell number in the ganglion cell layer (GCL) at 8 and 12 W was significantly lower in EAAC1 KO mice compared with WT mice (Figure 1c). In addition, the thickness of the inner retinal layer (IRL) was significantly decreased at 8 and 12 W in EAAC1 KO mice (Figure 1d). In edaravone-treated EAAC1 KO mice, the number of surviving neurons was significantly higher than that in control EAAC1 KO mice at 8 and 12 W (Figures 1b and c). In addition, edaravone treatment prevented the thinning of the IRL (Figure 1d). Because GCL contains cell types other than RGCs including displaced amacrine cells,^22^ we next performed retrograde labeling of RGCs with Fluoro-Gold (FG) and determined the effect of edaravone on RGC survival. Consistent with the results of cell counting in the GCL (Figure 1c), the RGC number in edaravone-treated mice was significantly increased compared with control mice treated with PBS (Figure 2).

**Figure 3:**
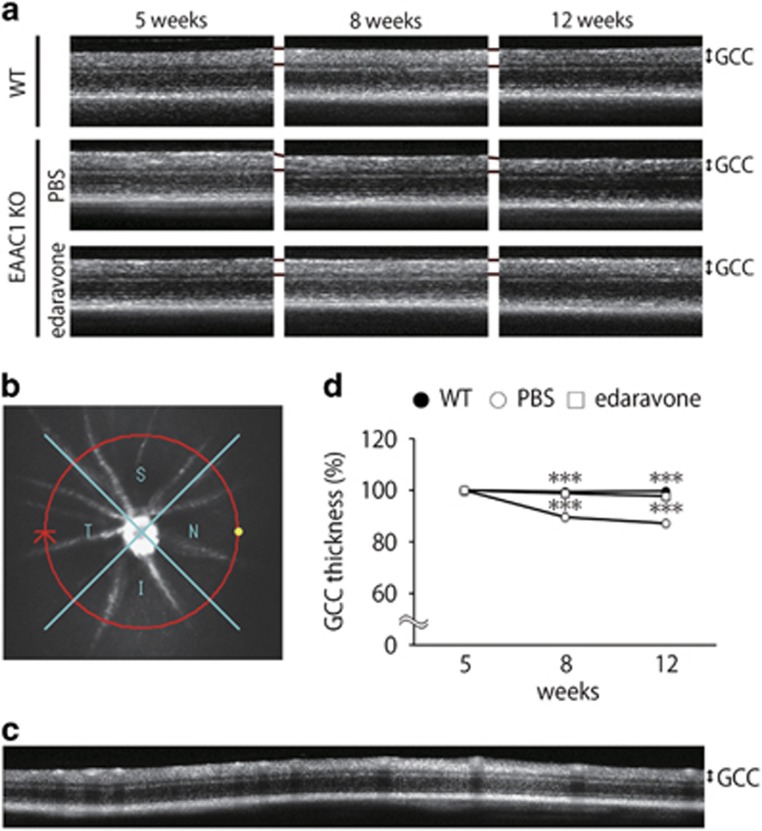
*In vivo* imaging of the retina in EAAC1 KO mice treated with edaravone. (**a**) OCT cross-sectional images of retinas at 5, 8 and 12 W. (b) An image of a circle centering around the optic nerve disk. (**c**) An OCT circular scan image captured from (**b**). (**d**) Longitudinal evaluation of the GCC thickness by a circular scan. The data are presented as means±S.E.M. of six samples for each experiment. ***P<0.001

We also visualized retinal layers in living mice using optical coherence tomography (OCT), a noninvasive imaging technique that can be used to acquire cross-sectional tomographic images of the retina *in vivo*.^19, 23^ The average thickness of the ganglion cell complex (GCC), which includes the nerve fiber layer, GCL, and inner plexiform layer, was significantly greater at 8 and 12 W in edaravone-treated EAAC1 KO mice compared with control EAAC1 KO mice (Figure 3a). For quantitative analysis, GCC was measured by scanning the retina in a circle centering around the optic nerve disk (Figure 3b), and the average GCC thickness was determined from acquired images (Figure 3c). GCC thickness at 8 and 12 W was significantly reduced in control mice, but it was almost unchanged in edaravone-treated mice (Figure 3d). These data indicate that edaravone treatment protects RGCs from NTG-like neurodegeneration.

### Edaravone ameliorates visual impairment in EAAC1 KO mice

**Figure 4:**
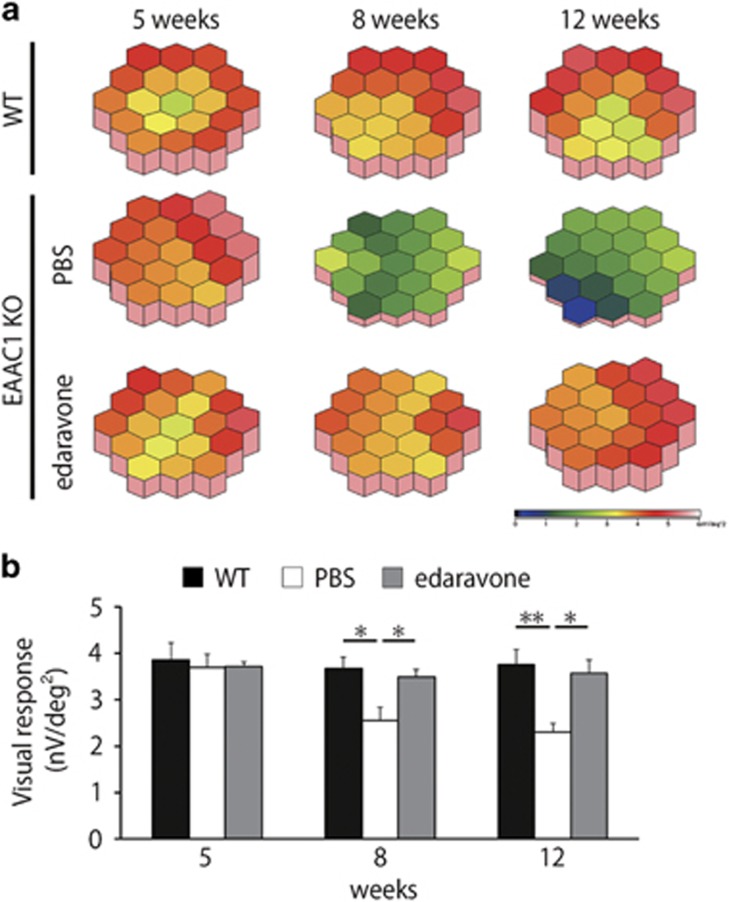
Effects of edaravone on visual response in EAAC1 KO mice. (**a**) Averaged visual responses of the second-order kernel demonstrated using three-dimensional plots. (**b**) Quantitative analysis of the visual response amplitude. The data are presented as means±S.E.M. of six samples for each experiment. *P<0.05, **P<0.01

To determine whether the histological observation of edaravone-mediated neuroprotection in EAAC1 KO mice reflects functional aspects, we examined visual function using multifocal electroretinogram (mfERG). We analyzed the second-order kernel component, which appears to be a sensitive indicator of inner retinal dysfunction and is impaired in glaucoma patients.^24, 25^ The response topography demonstrating the second-order kernel component revealed that the average visual responses were impaired in all visual fields in EAAC1 KO mice, but edaravone treatment ameliorated the deterioration in visual function (Figure 4). These results verify that the neuroprotective effects of edaravone on glaucomatous retinal degeneration in EAAC1 KO mice are functionally significant.

**Figure 5:**
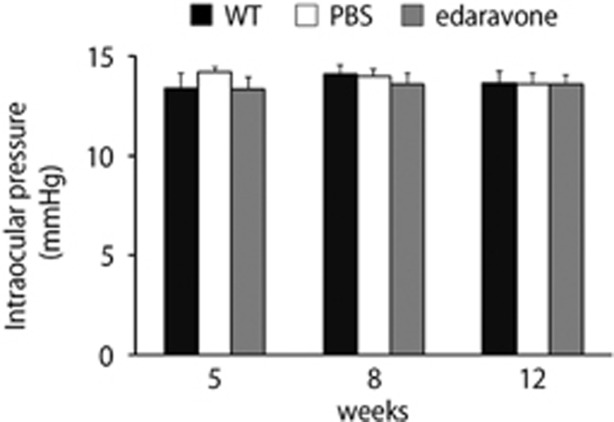
Effects of edaravone on intraocular pressure. The data are presented as means±S.E.M. of six samples for each experiment

We next examined the effects of edaravone on IOP. The previous study showed that IOP in EAAC1 KO mice is similar to that in WT mice.^7^ The IOP values of edaravone-treated EAAC1 KO mice were not significantly altered compared to those of control mice (Figure 5). These results suggest that edaravone prevents NTG-like pathology in EAAC1 KO mice and this neuroprotective effect is IOP-independent.

### Edaravone reduces the oxidative stress level in the EAAC1 KO mouse retina

**Figure 6:**
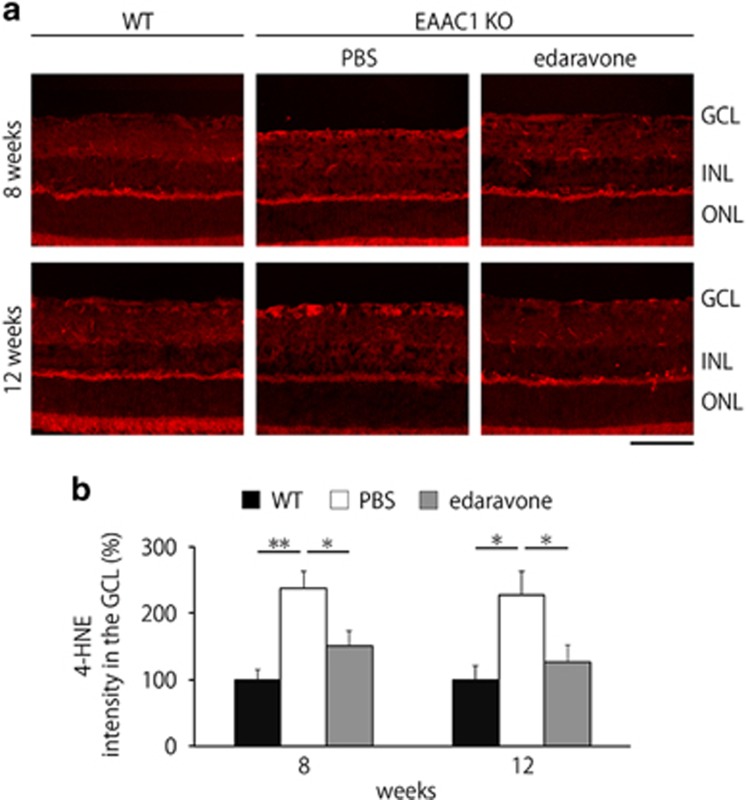
Edaravone reduces oxidative stress levels in the EAAC1 KO mouse retina. (**a**) Representative images of 4-HNE in the retina at 8 and 12 W. Scale bar: 100 *μ*m. (**b**) Quantitative analyses of (**a**). Data are normalized to the 4-HNE intensity at the GCL in control WT mice (100%). The data are presented as means±S.E.M. of six samples for each experiment. *P<0.05, **P<0.01

We next investigated potential mechanisms underlying edaravone-mediated neuroprotection. One of the major causes that is associated with glaucomatous retinal degeneration in EAAC1 KO mice is increased oxidative stress levels.^7, 19, 20, 26^ Therefore, we examined if edaravone treatment suppresses induction of oxidative stress in EAAC1 KO mice. For this purpose, we utilized 4-hydroxy-2-nonenal (4-HNE), which provides a reliable measure of oxidative stress.^20, 26^ 4-HNE was mainly observed in the GCL of EAAC1 KO mice, but it was hardly detected in WT mice or edaravone-treated EAAC1 KO mice at 8 and 12 W (Figure 6a). Quantitative analyses confirmed that the oxidative stress level in the GCL is significantly suppressed with edaravone treatment in EAAC1 KO mice (Figure 6b). These results suggest that edaravone prevents retinal degeneration in EAAC1 KO mice by suppressing the induction of oxidative stress in the retina.

## Discussion

In this study, we showed that edaravone prevents progressive RGC loss, thinning of the IRL and visual disturbances in EAAC1 KO mice without affecting IOP. To demonstrate these findings in the same animal, we utilized OCT and mfERG that permit *in vivo*, noninvasive and quantitative assessment of the changes in retinal morphology and function in EAAC1 KO mice. These techniques clearly visualized the therapeutic effects of edaravone and provide useful information in experimental animals as well as in clinical trials and management.

We previously reported that EAAC1 deficiency induces RGC loss mainly through oxidative stress.^7, 20, 26^ The main role of EAAC1 is to transport cysteine into RGCs as a precursor for neuronal glutathione synthesis, an important antioxidant.^27^ The concentration of plasma glutathione is decreased in glaucoma patients.^28, 29^ Consistently, suppression of oxidative stress exerts neuroprotective effects in EAAC1 KO mice.^10, 19, 20, 26^ We found that edaravone significantly suppressed the upregulation of 4-HNE, which is produced by lipid peroxidation during oxidative stress, in the GCL in EAAC1 KO mice (Figure 6). Increased 4-HNE modifies covalently several biomolecules containing amino groups, such as proteins, nucleotides and phospholipids, leading to disruption of its cellular functions. These results suggest that edaravone exerts antioxidative effects in the retina in EAAC1 KO mice.

Oxidative stress is an imbalance between the antioxidant system and the production of ROS. ROS includes O_2_^−^˙, ˙OH, H_2_O_2_ and singlet oxygen (^1^O_2_). In particular, the O_2_^−^˙ and ˙OH, which have an unpaired electron, are known as free radicals. Edaravone scavenges ˙OH, and inhibits lipid peroxidation and tyrosine nitration.^12, 13^ As edaravone suppresses lipid peroxidation both in water and lipid conditions,^12^ it may inhibit oxidative stress both at the cytosol, where water is the primary component, and the plasma membrane that has a fundamental structure of the phospholipid bilayer. In addition to antioxidative effects, edaravone has antiapoptotic effect. It shows a neuroprotective effect against ischemia/reperfusion brain injury through a Bax/Bcl-2-dependent antiapoptotic mechanism.^30^ We attempted to reveal the effects of edaravone on apoptotic pathways in EAAC1 KO mice, but unfortunately, we could hardly detect terminal deoxynucleotidyl transferase-mediated dUTP nick end-labeling-positive apoptotic cells, perhaps due to the mild progression of RGC degeneration. Thus, we could not quantify the effects of edaravone on the severity of apoptosis in the present study, but we believe it is possible that edaravone prevents retinal degeneration by modulating multiple apoptotic pathways in EAAC1 KO mice.

Recent studies have shown that oxidative stress has an important role in many ocular diseases, including glaucoma,^10, 29, 31, 32^ retinal detachment,^33^ diabetic retinopathy,^34^ age-related macular degeneration,^35^ retinitis pigmentosa^36^ and macular dystrophy.^37^ Consistently, intraperitoneal injection of edaravone was found to attenuate RGC death significantly in a streptozotocin-induced diabetes model.^34^ In addition, intravitreous and intravenous injections of edaravone significantly protected retinal neurons from glutamate neurotoxicity.^14^ Although further studies are required, these findings suggest a possibility that edaravone is useful for various retinal and optic nerve degenerative disorders.

We recently showed that every-other-day fasting (EODF), a form of caloric restriction, suppressed RGC death and retinal degeneration in EAAC1 KO mice without altering IOP.^20^ EODF upregulated brain-derived neurotrophic factor (BDNF), which induces neuroprotection, axonal outgrowth and neurogenesis, in the retina.^38, 39, 40^ Interestingly, BDNF signaling is activated by valproic acid and this pathway seems to play important roles in valproic acid-induced neuroprotection in GLAST KO mice.^41^ We have also reported that the orally active antagonist of angiotensin II type 1 receptor (AT1-R) suppressed Toll-like receptor 4 and lipopolysaccharide-induced inducible nitric oxide synthase expressions in EAAC1 KO mouse retina.^19^ Valproic acid and AT1-R antagonists are widely prescribed drugs for treatment of epilepsy and high blood pressure, respectively.^19, 41, 42^ These findings raise intriguing possibilities for the management of glaucoma by utilizing edaravone, a widely prescribed drug for the treatment of acute brain infarction and ALS, in combination with existing drugs for neuroprotection as well as conventional treatments to lower IOP.^19, 21, 38, 41^

## Materials and Methods

### Mice

Experiments were performed using EAAC1 KO mice (Miltenyi Biotec GmbH, Bergisch Gladbach, Germany)^7, 19, 43^ on a C57BL6 background in accordance with the Tokyo Metropolitan Institute of Medical Science Guidelines for the Care and Use of Animals.

### Drug administration

EAAC1 KO mice received daily intraperitoneal administration of vehicle (PBS) or edaravone (3 mg/kg; Mitsubisi Tanabe Pharma Co., Osaka, Japan) from 5 to 8 or 12 W. We selected the dose and route of edaravone administration based on previous studies that demonstrated its effects on retinal damages.^16, 34^

### Histologic and morphometric studies

Mice were perfused with Zamboni’s fixative (2% paraformaldehyde and 15% picric acid in 0.1 M phosphate buffer) at 5, 8 and 12 W. Eyes were enucleated and postfixed in 3% glutaraldehyde solution (3% glutaraldehyde, 9% formaldehyde, 37.5% ethanol and 12.5% acetic acid in distilled water) for 2 h. Paraffin-embedded retinal sections of 7 *μ*m thickness were cut through the optic nerve and stained with hematoxylin and eosin. The RGC number and the extent of retinal degeneration were quantified in two ways.^44^ First, the thickness of the IRL (between the internal limiting membrane and the interface of the outer plexiform layer and the outer nuclear layer) was analyzed. Second, in the same sections, the number of neurons in the GCL was counted from one ora serrata through the optic nerve to the other ora serrata.

### Retrograde labeling

Mice were deeply anesthetized with isoflurane (Intervet, Tokyo, Japan), placed on a stereotaxic frame and injected with 2 *μ*l of 2% FG (Fluorochrome LLC, Denver, CO, USA) dissolved in PBS into the superior colliculus.^45^ Ten days after FG application, mice were anesthetized, eyes were enucleated, and retinas were isolated for whole mount preparation. Retinas were fixed in 4% paraformaldehyde in 0.1 M PBS solution for 20 min, mounted on a glass slide with a mounting medium (Vectashield; Vector Laboratories, Burlingame, CA, USA), and the RGC density was examined with a fluorescent microscope. The excitation and emission wavelengths for FG were 323 nm and 620 nm, respectively. Three standard areas (0.04 mm^2^) of each retina at a point 0.1 mm from the optic disc were randomly chosen. FG-labeled cells were manually counted, and the mean number of RGCs per square millimeter was calculated.^45^

### Imaging acquisition of spectral-domain OCT

Spectral-domain OCT (RS-3000; Nidek, Aichi, Japan) examinations were performed at 5, 8 and 12 W. For fundus imaging, polymethyl methacrylate contact lenses optimal for mice (UNICON, Osaka, Japan) were placed on the corneas. Use of the lenses prevents anesthesia-induced cataract progression. A 60-D adaptor lens was placed on the objective lens of the Multiline OCT to focus on the mouse retina. All the line scan images were location matched, scanning vertically through the center of the optic nerve head at three disc diameter lengths above the optic nerve head.^19, 23^ The average thickness of GCC (between the internal limiting membrane and the interface of the inner plexiform layer and the inner nuclear layer) was measured. In this study, the maximum number of B-scans set by the manufacturer (50 for line scans) was used for averaging.

### mfERG

Mice at 5, 8 and 12 W were anesthetized by intraperitoneal injection of 87.5 mg/kg sodium pentobarbital. The pupils were dilated with 0.5% phenylephrine hydrochloride and 0.5% tropicamide. mfERGs were recorded using a VERIS 6.0 system (Electro-Diagnostic Imaging, Redwood City, CA, USA). The visual stimulus consisted of seven hexagonal areas scaled with eccentricity. The stimulus array was displayed on a high-resolution black and white monitor driven at a frame rate of 100 Hz. The second-order kernel, which is impaired in patients with glaucoma, was analyzed as previously reported.^7, 25, 44^

### IOP measurement

IOP was measured by a commercial rebound tonometer (TonoLab; Colonial Medical Supply, Franconia, NH, USA) in anesthetized mice as reported previously.^19, 44^ To minimize variation, the data were collected during a time window of 4–6 min after injection of the anesthetic, during which IOP plateaus. IOP was measured at 5, 8 and 12 W. As the 24 h IOP pattern in mouse eyes is biphasic, with IOP being the highest at ~2100 h,^46^ we examined IOP between 2000 h and 2300 h.

### Immunohistochemistry

Mice were perfused with Zamboni’s fixative at 8 and 12 W. Eyes were enucleated, postfixed in Zamboni’s fixative for 2 h and then transferred into a sucrose buffer (30% sucrose in a 0.1 M phosphate buffer) for cryoprotection. Retinal cryostat sections of 10 *μ*m thickness were prepared and examined by immunostaining using a 4-HNE mouse monoclonal antibody (0.2 *μ*g/mL; Japan Institute for the Control of Aging, Shizuoka, Japan). The intensity of 4-HNE at the GCL was analyzed using ImageJ (http://imagej.nih.gov/ij/; provided in the public domain by the National Institutes of Health, Bethesda, MD, USA).^41^

### Statistics

Data are presented as means±S.E.M. When statistical analyses were performed, the one-way ANOVA followed by a Turkey’s test or Student’s *t*-test was used. *P*<0.05 was regarded as statistically significant. JMP version 12.2.0 (SAS Institute Inc., Cary, NC, USA) was used for the statistical analyses.
